# Editorial: Neuropharmacological and neurotoxicological advances using *in vivo* non-mammalian model organisms

**DOI:** 10.3389/fnins.2023.1264745

**Published:** 2023-09-04

**Authors:** Lijie Xia, Meng Jin

**Affiliations:** ^1^Biology Institute, Qilu University of Technology (Shandong Academy of Sciences), Jinan, Shandong, China; ^2^Key Laboratory for Drug Screening Technology of Shandong Academy of Sciences, Jinan, Shandong, China

**Keywords:** neuropharmacology, neurotoxicity, disease modeling, drug discovery, neuroprotection

The tools and applications of *in vivo* non-mammalian models has rapidly advanced over the past few decades and opened new avenues for research. Given their advantages over traditional rodent models, there exists a great need to utilize and explore these model organisms within the field of neuroscience. This Research Topic, therefore, aims to highlight recent advances in neuropharmacology and neurotoxicity by modeling non-mammalian animal organisms, which exhibit similar functions and mechanisms with rodents during complicated neurological processes such as development, pathogenesis, etc.

In recent years, increasing research focused on promoting axonal regeneration, replenishing brain cells, and rebuilding lost pathways after nervous system injuries and neurodegenerative conditions. The ways of programmed cell death including apoptosis, necrosis, autophagy, and ferroptosis play a key role in the occurrence and development of neurodegenerative diseases, such as Alzheimer's disease (AD), Parkinson's disease (PD), and vascular dementia (Ji et al., 2022). Qi et al. explored the effects of Acrolein (ACR, a side-product of lipid peroxidation) on peripheral neuropathy using zebrafish model. The authors underlined excessive ferroptosis contributes to the impaired motor neuron development in ACR treated zebrafish embryos. They demonstrated that ACR could break cellular transition metal ion homeostasis, transition metal homeostasis, iron ion homeostasis, and iron ion transport. In addition, several factors involved in iron ion homeostasis pathways, such as fthl28, fthl29, fthl30, fthl31, and LOC100006428 are overexpressed. Therefore, ACR activated ferroptosis, interrupting homeostasis, which resulted in injured peripheral neurons. Notably, anti-ACR and anti-ferroptosis would be selected as promising therapeutic approaches for peripheral neuropathy. Autophagy, mitophagy as well as ubiquitin-proteasome system (UPS) dysregulation have pivotal roles in the clearance of damaged proteins and impaired organelles, which are highly associated with the onset of neurodegenerative diseases.

Wang et al. provided a thorough update of alleviating PD-like pathology in zebrafish. The authors revealed that the extract of Marigold *Calendula officinalis* L. (ECoL) protected dopaminergic neurons, central nervous system, and neural vasculature via the activation of autophagy pathway. Moreover, the PD-like locomotor retardation in zebrafish was obviously rescued. Specifically, the article discussed key genes involved in autophagy including *uchl1, pink1, parkin, ulk1b, ulk2, atg5, atg7, atg12, beclin1, ambra1a*, and *lc3b*. It suggested that ECoL obviously recovered the Pink1/Parkin-mediated mitophagy to clear dysfunctional mitochondria in zebrafish PD model. Meanwhile, ECoL could promote the formation of autophagic detached membrane and expedite the membrane separation in autophagy by up-regulating the expression of *ulk1b, ulk2, beclin1*, and *ambra-1a*. Furthermore, up-regulation of Atg genes (*atg5, atg7*, and *atg12*) and *lc3b* implied that the formation of mature autophagic vesicles was accelerated and autophagy restored to normal in ECoL-treated zebrafish. Collectively, zebrafish model offers a powerful tool for studying mechanisms of neurodegenerative conditions and exploring promising therapeutic drugs. This article would be very helpful to those interested in developing new strategies to regulate the autophagy pathway for the therapy of AD, PD, and other related neurodegenerative diseases.

In addition to neurodegenerative diseases induced cognitive dysfunction, anesthetics show similar effects. Tau protein, one of the key neuropathological hallmarks of AD, is important for microtubule assembly and maintaining its stability. Tau protein is mainly regulated by phosphorylation. Phosphorylated tau protein (p-Tau) is associated with cognitive dysfunction mediated by disrupting the stability of the microtubule structure (Nizynski et al., 2017). Hyperphosphorylated Tau accumulates in the form of neurofibrillary tangles (NFTs) in AD and triggers the degeneration or apoptosis of nerve cells, leading to cognitive function damage (Iqbal et al., 2005; Santacruz et al., 2005). AD-derived amyloid fibrils of Tau and Tau oligomers are also involved in neurodegenerative diseases via inhibiting the UPS. Chen et al. summarized the relationship between tau protein and cognitive impairments caused by different anesthetics. The authors found that anesthesia could cause tau phosphorylation, which is closely related to cognitive function. Thus, the mechanism underlying the effects of anesthetics on cognitive function via regulating tau protein has been widely studied. Ketamine can impair the synaptic function of the hippocampus and microtubule rupture by tau protein phosphorylation at Ser202/Thr205, Ser396, and sites Ser404. Similarly, propofol can increase p-Tau hippocampus and cortex, making neuronal cells re-enter into the cell cycle, thus leading to apoptosis. Hypoxic condition is triggered by propofol, which can activate p38, and then results in increased p-Tau. Midazolam is related to the continuous significant increase of p-Tau in the brain at AT8 (Ser202/Thr205), CP13, AT180, and Ser199 sites. Remimazolam injection can induce p-Tau at Ser202 and Thr232 in the cortex in a short time. However, long term administration of remimazolam reduces p-Tau at Ser396 and Thr205 by promoting the expression of PP2A. Morphine triggers p-Tau at Ser199, Ser202, Ser396, and Ser404, possibly through up-regulation of c-Jun NH2-terminal kinas (JNK) and Mitogen-activated protein kinases (MAPKs). Additionally, anesthesia-induced hypothermia leads to the inhibition of phosphatase activity as well as tau hyperphosphorylation. Sevoflurane can induce p-Tau at Ser202 and Thr205, trigger interleukin-6, decrease postsynaptic density protein 95 levels in the hippocampus, causing cognitive impairments. Dexmedetomidine increases p-Tau at Ser396 epitope with the potential to affect spatial reference memory. Taken together, anesthetic exposure exhibits significant differences in tau phosphorylation sites, which may be due to drug doses, administration speed, treatment methods, and even individual differences.

Refer to the mechanism of anesthetics, Liu et al. performed a further study to investigate the roles of GABAergic neurons in the hypothalamic tuberomammillary nucleus (TMN), with treatments of sevoflurane and propofol. In mammalian, the TMN innervated brain regions could regulate the sleep-wake cycle, due to the existence of the lateral hypothalamus (LH), ventrolateral preoptic nucleus (VLPO), basal forebrain (BF), and ventrolateral periaqueductal gray (vlPAG) (Yoshikawa et al., 2021). In zebrafish, GABAergic neurons in TMN project to the lateral habenula. The authors suggested that the activation of TMN GABAergic neurons weaken the anesthesia effect of sevoflurane and propofol. In contrast, the inhibition of TMN GABAergic neurons could enhance the depth and time of the anesthesia state in sevoflurane exposure instead of propofol. The findings from this article compensates for the limitations in the roles of GABAergic neurons in TMN.

This Research Topic highlights non-mammalian animal model, mainly zebrafish, from the broad range of emerging approaches that enable more scientific discoveries in molecular mechanism and neuropathology of neurodegenerative diseases. We want to share our excitement with the readers and witness novel directions that more methods will bring in the near future.

## Author contributions

MJ: Writing—review and editing. LX: Writing—original draft.
